# Using Team-Based Learning in an Online Synchronous Gerontology Course

**DOI:** 10.1093/geroni/igab046.387

**Published:** 2021-12-17

**Authors:** Leah Janssen

**Affiliations:** Scripps Gerontology Center, Oxford, Ohio, United States

## Abstract

Team-based learning (TBL) was chosen for its learner-centered approach to intentional engagement and purposeful application of course material in a cross-listed, upper-level gerontology class (i.e., Social Forces in Aging). Intedashboard, an online TBL platform, was utilized to support the online synchronous course, which is especially useful for its integration of class material, module assessments, peer/course evaluations, and dashboard display of live team activity. From the perspective of an emerging scholar, this symposia session will explore the application of TBL as a tool for developing teams, helping students personally connect with course material, and support inclusive teaching initiatives. More specifically, this presentation examines how a scaffolded TBL exercise on cumulative advantage/disadvantage, intersectionality, and social identities led to increased identification and awareness of students’ social location, and the perceived impacts on their later lives.

